# A Student-Led Mentorship Intervention to Enhance Podoconiosis Management Among Healthcare Workers and Village Health Teams in Western Uganda

**DOI:** 10.7759/cureus.100052

**Published:** 2025-12-25

**Authors:** Vicent Mwesigye, Joanita Berytah Tebulwa, Joseph Ngabirano, Trevor James Muhwezi, Racheal Ngasha, Daurice Patrice Najjingo, Hayidar Lubwama, Peter Chris Kawungezi, Grace Kitunzi Mulyowa, Edgar Mulogo

**Affiliations:** 1 Department of Medical Laboratory Science, Mbarara University of Science and Technology, Mbarara, UGA; 2 Department of Nursing, Mbarara University of Science and Technology, Mbarara, UGA; 3 Department of Community Health, Mbarara University of Science and Technology, Mbarara, UGA; 4 Department of Internal Medicine, Mbarara University of Science and Technology, Mbarara, UGA; 5 Department of Dermatology, Mbarara University of Science and Technology, Mbarara, UGA

**Keywords:** community health, foot hygiene, lymphedema, neglected tropical diseases, non-filarial elephantiasis, podoconiosis, uganda, village health teams

## Abstract

Podoconiosis is a neglected tropical, nonfilarial lymphedema resulting from prolonged barefoot exposure to irritant volcanic soils. Uganda is among the affected countries, with Kamwenge District documented as endemic. Persistent gaps in knowledge, prevention practices, and access to care contribute to a sustained disease burden. A multidisciplinary team from Mbarara University of Science and Technology conducted a three-day community-based situation analysis and mentorship program in Kamwenge District from October 3 to October 7, 2022. The team included undergraduate and postgraduate trainees in medical laboratory sciences, medicine, nursing, public health, and dermatology under faculty supervision. Following approval from district authorities, 50 community participants were engaged: 30 podoconiosis patients, 12 Village Health Team (VHT) members, five local council leaders, and seven patient caretakers. Activities included free clinical consultations, structured health education, psychosocial counseling, and hands-on demonstrations of limb hygiene, bandaging, appropriate footwear use, and the application of topical antibacterial or antifungal creams. Substantial knowledge gaps related to podoconiosis causation, prevention, and long-term management were identified among both patients and VHTs. Patients described significant stigma, chronic pain, and functional disability, which were also directly observed during mentorship interactions. Practical demonstrations of limb hygiene and bandaging techniques were positively received, and VHTs expressed strong willingness to integrate these practices into routine community follow-up. However, resource limitations, particularly inadequate footwear and medical supplies, emerged as major barriers to sustained self-care. Podoconiosis remains preventable through proper foot hygiene, sustained health education, use of protective footwear, and community empowerment. However, significant knowledge gaps and misconceptions persist in endemic areas of Kamwenge District. The use of multidisciplinary, community-engaged mentorship models can enhance capacity for early detection, self-care, and stigma reduction. Larger district-wide studies are warranted to better characterize the epidemiology and guide sustainable interventions.

## Introduction

Podoconiosis is a chronic, progressive, noninfectious lymphedema of the lower limbs, primarily affecting impoverished populations exposed to irritant volcanic soils. It is characterized by bilateral, asymmetrical swelling of the legs due to an abnormal inflammatory response to mineral particles penetrating the skin of barefoot individuals [1-3]. The disease disproportionately impacts subsistence farming communities in tropical highland regions of Africa, Asia, and Latin America, where barefoot walking is common and soil exposure is prolonged [4].

In Uganda, podoconiosis has been documented in several highland districts, including Kamwenge, where environmental exposure, low socioeconomic status, and inadequate protective footwear contribute to the ongoing disease burden [2,5]. Estimates suggest that hundreds of individuals may be affected in Kamwenge alone, yet formal surveillance remains limited.

Mbarara University of Science and Technology (MUST), through its Community-Based Education, Research and Service (COBERS) model, routinely partners with rural districts to strengthen primary health care, especially for neglected conditions. In line with this mission, a multidisciplinary MUST team conducted a podoconiosis-focused mentorship and outreach intervention in Kamwenge District. The objective was to analyze the local situation, assess knowledge and care practices, and build the capacity of Village Health Teams (VHTs) and community members through targeted training.

## Materials and methods

This community-based intervention study was conducted from October 3 to October 7, 2022, in Busiriba Sub-county of Kamwenge District, Uganda. Prior to implementation, formal authorization was obtained from the District Chairperson, District Health Officer, and sub-county leadership. Local council leaders supported community mobilization and ensured that all participants provided verbal consent.

The intervention team comprised three undergraduate students (medical laboratory sciences, medicine, and nursing), two postgraduate trainees (public health and dermatology), and faculty mentors from dermatology and medical laboratory sciences. Team members collaborated using a multidisciplinary approach to address medical, psychosocial, and community engagement needs.

Fifty participants were purposively enrolled to ensure inclusion of key stakeholders directly involved in podoconiosis care and community support, including 30 diagnosed patients, 12 VHT members, five council representatives, and three patient caretakers. This sampling approach was chosen to capture context-specific experiences, knowledge gaps, and care practices relevant to the mentorship intervention. As such, the findings are descriptive and exploratory and are not intended to be statistically generalizable beyond the study setting. Data were collected through interactive educational sessions, clinical encounters, direct observation of limb conditions, and community discussions. No identifiable patient information was collected.

Health education sessions were conducted at Busiriba Health Centre II and addressed the pathophysiology, causes, risk factors, and prevention of podoconiosis. Emphasis was placed on the importance of consistent footwear use, soil avoidance strategies, daily foot hygiene, and early recognition of complications such as acute dermatolymphangioadenitis (ADLA). Psychosocial challenges, including stigma, isolation, and reduced quality of life, were discussed openly to encourage support-seeking and community cohesion.

**Figure 1:**
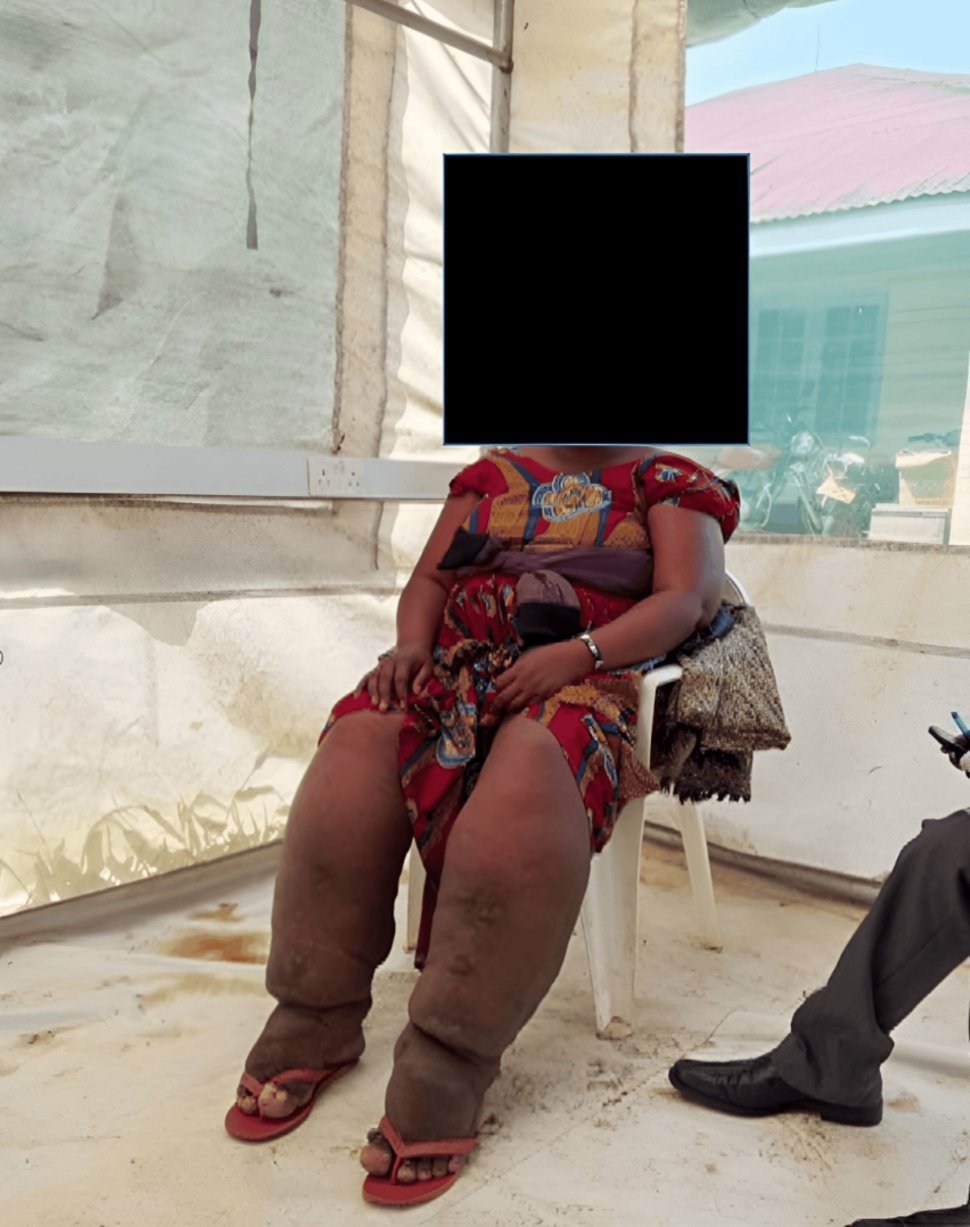
A participant engaged in a lower-limb care training session for podoconiosis

Practical demonstrations were led by dermatology trainees and faculty and included proper limb washing using soap and clean water, drying techniques, application of emollients, bandaging for lymphedema management, and appropriate use of topical antibacterial or antifungal agents. Participants were encouraged to practice these techniques to ensure proficiency. Free medical consultations were provided for podoconiosis patients and other community members, including dermatologic assessment, screening for comorbid conditions, mental health support, and individualized counseling, as shown in Figure 1.

## Results

A total of 50 participants took part in the intervention, including 30 podoconiosis patients, 12 VHT members, five local council representatives, and seven patient caretakers. Among the podoconiosis patients, most reported longstanding lower-limb swelling (24/30, 80%) and frequent episodes of ADLA (18/30, 60%). Knowledge regarding the etiology and prevention of podoconiosis was limited, with 20 patients (66.7%) demonstrating poor understanding of the disease and 10 (33.3%) attributing their condition to witchcraft or hereditary curses, reflecting persistent misconceptions documented in other endemic regions.

Prior to the intervention, VHT members demonstrated limited capacity to recognize podoconiosis and provide guidance on long-term management. Following the training, most VHTs expressed improved confidence in key areas, including assisting patients with basic limb hygiene (10/12, 83%), recognizing complications such as ADLA (9/12, 75%), and delivering community health education on podoconiosis prevention and care (10/12, 83%). These findings suggest that targeted, hands-on training can rapidly enhance community health worker competency in managing podoconiosis.

Interactive demonstrations of foot washing, drying, bandaging, and application of topical antibacterial or antifungal creams were well received. All participants practiced these techniques under supervision, and approximately 70% of patients and VHTs expressed intent to incorporate them into their daily routines. Emphasis on routine limb hygiene and the importance of protective footwear was reinforced throughout the sessions. Despite high engagement, several structural and resource-related barriers were identified. Specifically, 60% of patients reported a lack of access to soap for daily foot hygiene, 50% lacked adequate bandaging materials or emollients, and 80% had limited access to protective footwear. These barriers represent significant challenges to consistent self-care and indicate the need for interventions that address both education and material support.

High levels of psychosocial distress were also observed among patients, including stigma (18/30, 60%), low self-esteem (15/30, 50%), and social exclusion (12/30, 40%). These issues were evident during clinical consultations and community discussions, highlighting the broader social and emotional burden of podoconiosis in affected communities. These findings are summarized in Table 1.

**Table 1:** Summary of participant characteristics, knowledge, skills, practices, barriers, and psychosocial factors ADLA, acute dermatolymphangioadenitis; VHT, Village Health Team

Domain	Indicator	Number of participants	Percentage (%)
Participants (n = 50)	Podoconiosis patients	30	60
	VHT members	12	24
	Local council representatives	5	10
	Patient caretakers	3	6
Patient clinical characteristics	Longstanding lower limb swelling	24	80
	Frequent ADLA episodes	18	60
Patient knowledge and misconceptions	Limited knowledge of podoconiosis etiology	20	66.7
	Attributed disease to witchcraft/hereditary curses	10	33.3
VHT skills (post-training)	Confident in assisting with limb hygiene	10/12	83
	Confident recognizing complications (ADLA)	9/12	75
	Confident delivering community health education	10/12	83
Self-care practices	Expressed intent to incorporate hygiene practices	35	70
Resource barriers (patients)	Lack of soap	18	60
	Lack of bandaging materials/emollients	15	50
	Limited access to protective footwear	24	80
Psychosocial observations	Experienced stigma	18	60
	Low self-esteem	15	50
	Social exclusion	12	40

## Discussion

The findings from this intervention underscore the persistent burden of podoconiosis in Kamwenge District and the multifaceted challenges faced by affected communities. Similar to studies in Ethiopia, Rwanda, and Cameroon, misconceptions about causation persist, contributing to stigma and delays in seeking care [6-10]. The effectiveness of consistent footwear use, foot hygiene, and community-based lymphedema management has been well documented [4,11,12]; however, adoption remains low in Uganda.

The integration of undergraduate and postgraduate trainees into community-engaged mentorship aligns with best-practice models for strengthening primary health care in resource-limited settings [13]. This approach proved valuable in building VHT capacity and fostering trust between communities and health workers. As highlighted in previous field-based podoconiosis programs, decentralizing care to community health workers improves adherence and outcomes [11,14].

Psychosocial challenges emerged prominently during discussions with patients. Stigma has been shown to significantly reduce quality of life, limit social participation, and worsen mental health among individuals with podoconiosis [14,15]. Addressing these concerns requires sustainable community education, patient support groups, and leadership involvement strategies, as implemented during this outreach.

However, structural barriers such as poverty and limited access to footwear remain significant. Protective footwear distribution programs have demonstrated cost-effective reductions in podoconiosis incidence in high-risk settings [6], and similar interventions are needed in Uganda. The short duration of this outreach limited its overall reach, suggesting the value of longitudinal community engagement and district-wide mapping studies.

This study had several limitations that may affect the interpretation of its findings. The short duration of the outreach program limited its reach, and longer-term engagement is needed to assess lasting impacts. Additionally, the cross-sectional design means that conclusions about the long-term effectiveness of footwear use, foot hygiene, and community-based management may be premature. Structural barriers, such as poverty and lack of access to footwear, likely reduced the intervention’s effectiveness, and without protective footwear programs, assessing their full impact in Uganda is challenging. The psychosocial impact of stigma was significant but not fully addressed, indicating the need for more comprehensive strategies. Finally, while integrating trainees into the mentorship model helped build trust and capacity, its effectiveness may vary across different contexts, requiring further research on the sustainability and scalability of such programs in resource-limited settings.

## Conclusions

Podoconiosis remains a preventable cause of chronic lymphedema in Kamwenge District. The findings highlight persistent misconceptions, limited access to preventive supplies, and the need for improved community-based management. Multidisciplinary mentorship models can strengthen skills among VHTs, enhance patient self-care, and reduce psychosocial distress. Broader epidemiological studies are necessary to guide large-scale intervention planning and resource allocation.

## References

[1] Price EW, Frommel D (2019). Podoconiosis (nonfilarial elephantiasis). The Ecology of Health and Disease in Ethiopia.

[2] Kihembo C, Masiira B, Lali WZ (2017). Risk factors for podoconiosis: Kamwenge District, Western Uganda, September 2015. Am J Trop Med Hyg.

[3] Davey G, Newport M (2007). Podoconiosis: the most neglected tropical disease?. Lancet.

[4] Hailu M, Chea N, Ali MM, Hailu M (2023). Determinants of Podoconiosis in Bensa District, Sidama Region, Ethiopia: a case control study. PLoS Negl Trop Dis.

[5] Masete I, Simpson H, Matwale G (2024). Podoconiosis in Uganda: prevalence, geographical distribution and risk factors. Trans R Soc Trop Med Hyg.

[6] Deribe K, Cano J, Trueba ML, Newport MJ, Davey G (2018). Global epidemiology of podoconiosis: a systematic review. PLoS Negl Trop Dis.

[7] Engdawork K, Tadele G, Nahar P, Davey G, Zaman S (2024). The effect of contextual factors on a health intervention against podoconiosis in Ethiopia. Front Trop Dis.

[8] Bikorimana JP, Davey G, Mukabera J, Shahaduz Z, Mugume PJ, Nahar P (2024). Uncovering intersecting stigmas experienced by people affected by podoconiosis in Nyamasheke district, Rwanda. PLoS Negl Trop Dis.

[9] Wanji S, Tendongfor N, Esum M (2008). Elephantiasis of non-filarial origin (podoconiosis) in the highlands of north-western Cameroon. Ann Trop Med Parasitol.

[10] Troost MA (2019). The impact of family-based approaches aimed at prevention and sustainable self-management of
disabilities on persons affected by lymphatic filariasis (LF) and podoconiosis and their family members in the
Amhara region, Ethiopia. A mixed methods study [Internship report]. https://disabilitystudies.nl/sites/default/files/beeld/publicaties/internship_report_meike_troost_final.pdf.

[11] Negussie H, Molla M, Ngari M (2018). Lymphoedema management to prevent acute dermatolymphangioadenitis in podoconiosis in northern Ethiopia (GoLBeT): a pragmatic randomised controlled trial. Lancet Glob Health.

[12] Kansiime C, Rutebemberwa E, Mugisha A, Mugisha S, Asiimwe BB, Rwego IB, Kiwanuka SN (2014). Determinants of patients' choice of provider in accessing brucellosis care among pastoral communities adjacent to lake Mburo National Park in Kiruhura District, Uganda. PLoS ONE.

[13] Enbiale W, Deribe K (2025). Podoconiosis: a comprehensive clinical review and strategies for control and elimination. Clin Exp Dermatol.

[14] Tora A, Franklin H, Deribe K, Reda AA, Davey G (2014). Extent of podoconiosis-related stigma in Wolaita Zone, Southern Ethiopia: a cross-sectional study. Springerplus.

[15] Abiso TL, Kerbo AA, Woticha EW, Koyira MM (2024). Stigma related to podoconiosis in Ethiopia: a systematic review. Front Trop Dis.

